# Target bronchus determination in giant emphysematous bullae: A case report

**DOI:** 10.1097/MD.0000000000043003

**Published:** 2025-06-20

**Authors:** Lecong Ouyang, Weidong Zhang, Zeqiang Wang

**Affiliations:** a Department of Respiratory and Critical Care Medicine, Hunan Provincial People’s Hospital/The First Affiliated Hospital of Hunan Normal University, Changsha, Hunan, China.

**Keywords:** bronchoscopic volume reduction, chronic obstructive pulmonary disease, giant emphysematous bullae, percutaneous puncture, target bronchus

## Abstract

**Rationale:**

Giant emphysematous bullae (GEB) in chronic obstructive pulmonary disease cause severe respiratory compromise. While surgical resection is standard, bronchoscopic volume reduction is crucial for surgically ineligible patients. Accurate target bronchus identification remains challenging with conventional imaging.

**Patient concerns:**

A 67-year-old male with chronic obstructive pulmonary disease and right lung GEB presented with severe dyspnea (modified Medical Research Council score 4), hypercapnia (partial pressure of carbon dioxide: 45 mm Hg), and markedly limited exercise tolerance (6-minute walk distance: 62 m). He required home noninvasive ventilation and was deemed unfit for surgery due to critically impaired lung function (Forced expiratory volume in 1 second: 0.36 L, 12.2% predicted).

**Diagnoses:**

Preoperative high-resolution computed tomography (CT) and 3D reconstruction localized the target bronchus to the right middle lobe. However, percutaneous aspiration and drug injection via drainage tube revealed misalignment, prompting reidentification of the target bronchus in the posterior segment of the right upper lobe.

**Interventions:**

CT-guided percutaneous GEB volume reduction was performed, involving air extraction and intrabullous injection of erythromycin lactobionate. Subsequent selective bronchial occlusion of the posterior right upper lobe segment via bronchoscopic autologous blood and thrombin injection was conducted. Continuous negative-pressure drainage was maintained post-procedure.

**Outcomes:**

Follow-up CT at 6 months confirmed complete GEB closure. Dyspnea improved significantly (modified Medical Research Council score 3), exercise capacity increased (6-minute walk distance: 220 m), and ventilator use was discontinued. No complications or recurrence were observed during follow-up.

**Lessons:**

Percutaneous aspiration and drug injection refine target bronchus identification when imaging yields ambiguous results, enhancing precision for subsequent bronchoscopic interventions. This strategy minimizes reliance on endobronchial valves, reducing costs and procedural complexity. Larger studies are needed to validate long-term efficacy, but this approach offers a promising minimally invasive alternative for high-risk patients.

## 1. Introduction

Bullae refer to thin-walled air sacs in the lungs that exceed 1 cm in diameter and are commonly associated with chronic obstructive pulmonary disease. As the condition progresses, these bullae can evolve into giant emphysematous bullae (GEB), which typically occupy more than one-third of the volume of a hemithorax.^[1]^ GEB compresses the surrounding lung tissue and disrupts gas exchange, leading to breathing difficulties during physical activity. Although surgery remains the primary treatment for GEB, it requires patients to meet specific health criteria, and some individuals may be unable to tolerate the associated risks.^[2,3]^

Endobronchial valve (EBV) placement has emerged as a widely accepted alternative treatment option.^[4–7]^ Accurate localization of the target bronchus with the GEB is crucial for successful intervention. Traditionally, we used high-resolution computed tomography (HRCT) scans and 3-dimensional reconstruction techniques to identify the target bronchus with GEB.^[8]^ However, GEB compression distorts the aerated bronchial tree, altering bronchial direction and orifice position, thereby complicating target bronchus identification on HRCT images. Our study on percutaneous GEB volume reduction found that suction combined with intrabullous drug injection via a drainage tube significantly improved target bronchus localization. We present a case of successful percutaneous GEB volume reduction with favorable clinical outcomes.

## 2. Case presentation

A 67-year-old male patient with a 2-year history of GEB in the right lung and an 11-year history of COPD presented to our department. The patient had been regularly using inhaled medications and required home noninvasive ventilator support owing to the severity of his condition. Prior to our intervention, the patient’s lung function tests revealed a forced expiratory volume in 1 second (FEV_1_) of 0.36 L, which is 12.2% of the predicted value, and a forced vital capacity of 1.40 L, equating to 36.7% of the predicted value. The 6-minute walk test distance was merely 62 m, indicative of his severely limited exercise tolerance. Blood gas analysis revealed hypercapnia with a partial pressure of carbon dioxide of 45 mm Hg. The St. George’s Respiratory Questionnaire total score was 86, and the modified Medical Research Council score for dyspnea was 4, both reflecting poor quality of life and severe dyspnea. Due to poor lung function and hypercapnia, the thoracic surgeon deemed conventional surgery unsuitable. Ultimately, the patient was transferred to our department and underwent percutaneous GEB volume reduction. This minimally invasive technique reduces the size of giant bullae and improves pulmonary function. The procedure consists of 3 key steps.

**Figure 1.**
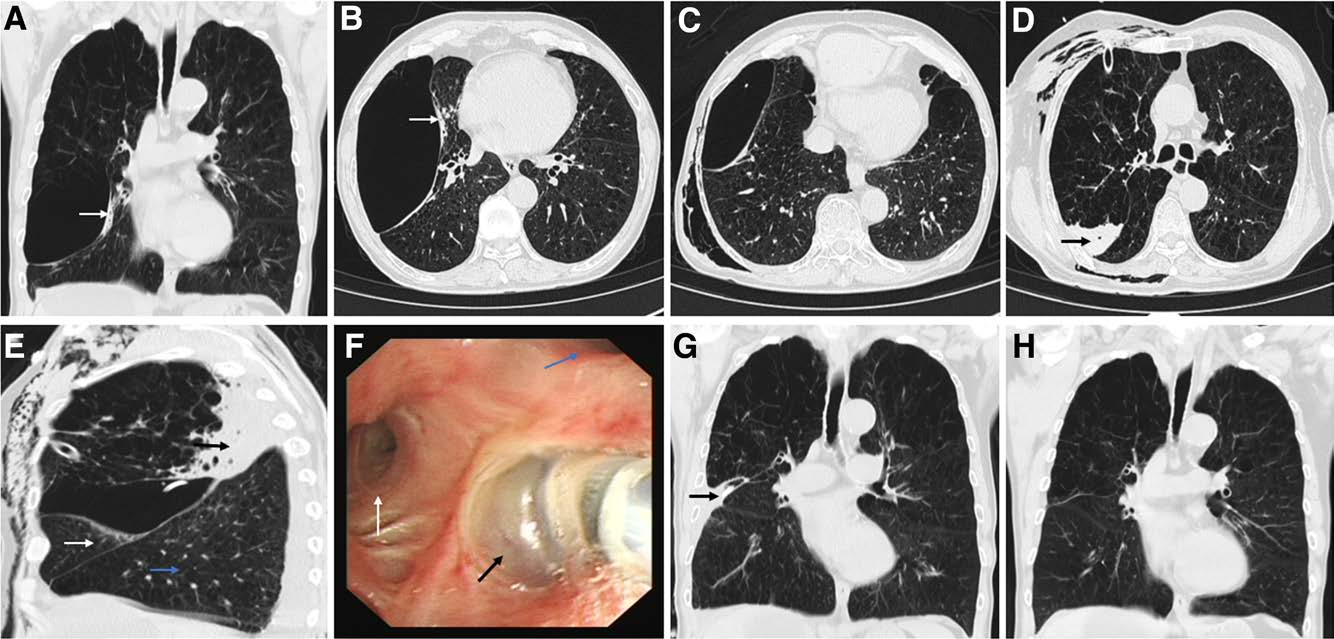
Procedure of GEB volume reduction and confirmation of the GEB target bronchus in the Patient. (A, B) Before treatment, GEB occupied the right middle and right lower lungs, and the radiologist evaluated the target bronchus as the lateral segment of the right middle lobe (white arrow). (C) Under the guidance of CT, a central venous catheter was inserted into the GEB, air was pumped through the drainage tube, and medication was injected. The GEB atrophied to the right upper lobe. (D) Drug deposition in the posterior segment of the right upper lobe (black arrow). (E) Anatomical demarcation reveals: right middle lobe (white arrow), right lower lobe (blue arrow), and posterior segment of right upper lobe (black arrow). Drug deposition and drug-related pneumonia are visible in the posterior segment of the right upper lobe, with reduced GEB and drainage tubes seen in the surrounding area. (F) Bronchoscopic visualization of the right upper lobe bronchial orifice demonstrates: apical segment (blue arrow), anterior segment (white arrow), and posterior segment (black arrow). Using a 3-chamber lithotomy balloon, autologous blood was injected into the posterior segment of the right upper lobe. (G) Before discharge, CT was reexamined, indicating that the GEB was nearly closed (black arrow). (H) No recurrence of pulmonary bullae was observed during a follow-up examination 6 months after the procedure. CT = computed tomography, GEB = giant emphysematous bullae.

Step 1: CT-guided GEB Catheterization. The radiologist analyzed the preoperative HRCT results and identified the target bronchus associated with the GEB in the lateral segment of the right middle lobe (Fig. 1A, B). Using the Seldinger technique under CT guidance,^[9]^ a 16-gauge single-lumen central venous catheter was advanced into the GEB (Fig. 1C). A 3-way connector was attached to the proximal end of the catheter, and a 50 mL syringe was used to aspirate air through the connector. During the procedure, patients required close monitoring. If dyspnea worsened, air aspiration was immediately discontinued. The aspirated volume was controlled between 1000 and 2000 mL. Post-aspiration CT imaging was performed to assess the degree of GEB reduction, post-deflation positional changes, and catheter depth (Fig. 1C). Pneumothorax monitoring was essential during the process, and the drainage tube depth was adjusted as needed.

Step 2: Erythromycin Lactobionate Injection and Negative-Pressure Drainage. A dose of 1 g erythromycin lactobionate (dissolved in 50 mL of 50% glucose) was injected into GEB through the central venous catheter. The 3-way connector was closed immediately after injection, and CT imaging was performed 10 minutes later to observe drug deposition patterns and assess for delayed pneumothorax. Post-procedure, the patient underwent continuous ECG monitoring and vital sign surveillance. Follow-up CT revealed contraction of the GEB toward the right upper lobe, with drug deposition localized to the posterior segment of the right upper lobe (Fig. 1C, D). Gas accumulation in the right thoracic cavity was noted, prompting immediate placement of a thoracic drainage tube (Fig. 1D). After 4 hours, a 3-chamber closed thoracic drainage system was initiated for continuous negative-pressure suction (−12 cm H_2_O to −18 cm H_2_O) for 3 to 5 days. Four days post-intervention, repeat chest CT demonstrated bulla size reduction in the right upper lobe and drug-related pneumonia in the posterior segment (Fig. 1E). No drug deposition or pneumonia was observed in the other right lung regions. These findings confirmed the posterior segment of the right upper lobe as the GEB-associated target bronchus, suggesting potential inaccuracies in the initial radiological assessment.

Step 3: Bronchoscopic Selective Bronchial Occlusion. Five days after the GEB puncture, we inserted a 3-chamber lithotomy balloon via bronchoscopy into the posterior segment of the right upper lobe (Fig. 1F) and simultaneously injected 30 mL of autologous blood mixed with 10 mL thrombin solution (100 U/mL). Post-procedural drainage was maintained. No gas output was observed from the GEB or pleural drainage after 8 days. A follow-up chest CT revealed near-complete closure of the GEB (Fig. 1G). The patient demonstrated significant improvement in dyspnea and exercise tolerance compared to preoperative status, eliminating the previous requirement for home noninvasive ventilator support.

Six-month follow-up CT confirmed complete GEB closure (Fig. 1H). Dyspnea severity decreased (modified Medical Research Council score from 4 to 3), with increased exercise capacity (6-minute walk distance improved from 150 to 220 m). No complications or disease recurrence were observed. Detailed clinical parameters are summarized in Table 1.

**Table 1:** Comparison of lung function, blood gas analysis, dyspnea, and quality of life scores in GEB patients before surgery, before discharge and 6 months after surgery.

Variables	Before surgery	Before discharge	Follow-up at 6 mo after surgery
FEV_1_ (l)	0.36	0.62	0.66
FEV_1_ (% pred)	12.2	20.8	23.2
FVC (l)	1.40	1.74	1.86
FVC (% pred)	36.7	45.6	49.0
RV (l)	8.60	6.23	6.02
RV (% pred)	236.2	171.0	164.5
TLC (l)	9.20	7.25	7.18
TLC (% pred)	137.0	108.0	107.0
6MWT (m)	62	210	220
PaCO_2_ (mm Hg)	45	40	39
PaO_2_/FIO_2_	222	300	322
Total SGRQ score	86	64	62
mMRC score	4	3	3

%pred = %predicted, 6MWT = 6-minute walking testing, FEV_1_ = forced expiratory volume in 1 second, FIO_2_ = fraction of inspired oxygen, FVC = forced vital capacity, mMRC = modified Medical Research Council, PaCO_2_ = partial pressure of carbon dioxide, PaO_2_ = partial pressure of oxygen, RV = residual volume, SGRQ = St. George’s Respiratory Questionnaire, TLC = total lung capacity.

## 3. Discussion

The pathogenesis of GEB is closely associated with the progression of COPD. Core mechanisms include smoking, α1-antitrypsin deficiency, intravenous drug use, cannabis and cocaine consumption, sarcoidosis, and autoimmune disorders.^[10,11]^ These pathological processes lead to the destruction of alveolar walls, the loss of elastic recoil, and the coalescence of adjacent damaged alveoli, ultimately forming large air-filled cavities with no gas-exchange capacity.^[12]^ Notably, bullae predominantly affect upper lung lobes (particularly the apical and posterior segments),^[13,14]^ which may correlate with gravitational stress and heterogeneous emphysema distribution.^[15]^ Due to their superior positioning, these bullae may exert compressive effects on healthier lower lobe parenchyma, resulting in restricted ventilation and perfusion that exacerbates functional impairment.

Patients with GEB typically do not respond to medical treatment, making surgical removal the preferred option because of its potential to significantly improve lung function and exercise tolerance.^[8,16]^ However, studies have shown that in patients with a preoperative FEV_1_% < 35% who also present with hypoxemia and hypercapnia, postoperative functional recovery is often unsatisfactory, even after GEB resection.^[17]^ The primary reasons include: Patients with severely reduced FEV_1_ have minimal residual lung capacity to compensate post-resection. Even after removing the GEB, the remaining emphysematous lung parenchyma often fails to expand adequately, resulting in suboptimal functional improvement. As noted in reference,^[16]^ postoperative complications (e.g., persistent air leaks, arrhythmias) occur more frequently in high-risk patients, prolonging recovery and increasing morbidity. In such cases, the risks of surgery often outweigh the benefits. Nakahara et al^[17]^ demonstrated that surgical outcomes correlate strongly with preoperative lung function. Patients with FEV_1_% < 35% showed limited functional recovery, underscoring the need for alternative approaches in this subgroup. Thus, while surgery remains effective for select patients with preserved lung function, our case emphasizes the value of minimally invasive techniques (e.g., percutaneous and bronchoscopic volume reduction) for high-risk, surgically ineligible individuals. These approaches mitigate procedural risks while achieving meaningful clinical improvement, as demonstrated in our patient’s outcome.

Bronchoscopic and percutaneous GEB volume-reduction techniques have emerged as standard alternative treatments for patients with GEB who are ineligible for surgery.

Selecting the correct segmental bronchus for EBV placement in giant emphysematous bullae GEB can be challenging. Conventional imaging techniques, such as high-resolution HRCT and 3D reconstruction, are often used to identify the target bronchus. However, the compression and distortion of adjacent bronchial structures by GEB may obscure anatomical relationships, leading to misidentification of the target bronchus, as demonstrated in our case, where initial HRCT localization discrepancies were corrected via percutaneous aspiration and drug injection, although such cases are relatively rare. Additionally, GEB may alter bronchial orientation or collapse aerated pathways, complicating preoperative planning. Advanced navigation tools (e.g., electromagnetic or virtual bronchoscopy) may improve accuracy, but their efficacy in GEB cases remains understudied.^[18,19]^ Furthermore, the absence of interlobar collateral ventilation must be confirmed for EBV success, requiring adjunctive assessments like the Chartis system.^[7]^ These technical and anatomical challenges highlight the need for multimodal approaches to refine target bronchus identification.

Meta-analytical evidence indicates that the success of EBV therapy is highly dependent on achieving complete isolation of the target lung region.^[20]^ In bronchoscopic lung volume reduction, the subgroup of patients with intact interlobar fissures (a surrogate marker suggesting absence of collateral ventilation^[21]^) demonstrated significant improvements in FEV₁ at 6 months and 1 year post-procedure, whereas those with incomplete fissures showed no substantial benefit. This conclusion similarly applies to GEB treatment. For instance, Tian et al^[7]^ utilized the Chartis system to evaluate collateral ventilation status and performed EBV therapy in 5 patients with GEB patients: Two collateral ventilation-negative patients exhibited an increase in FEV₁% predicted from 27% to 32% at 1 month postoperatively, with 1 right middle lobe GEB patient maintaining functional improvement during 6-month follow-up; whereas no clinical or radiological improvements were observed in the 3 collateral ventilation-positive patients. Therefore, GEB patients with persistent collateral ventilation may not be suitable candidates for EBV therapy due to the inability to achieve effective target zone collapse.

As indicated by relevant studies,^[22,23]^ patients with excessive airway secretions may compromise the efficacy of EBV therapy. After EBV placement, secretions are prone to accumulating on the valve surface. Particularly in patients with a heavy and viscous airway secretion burden, the risk of valve obstruction caused by secretions is increased. This can lead to improper valve closing or opening, thereby undermining the therapeutic effect and potentially necessitating valve reinsertion. Consequently, EBV replacement may not be suitable for patients with abundant airway secretions.

Takizawa et al^[24]^ pioneered a percutaneous bulla drainage technique involving CT-guided insertion of a 6F balloon catheter via GEB puncture, connected to a negative-pressure water-seal system for continuous drainage. The protocol included administering the immunostimulant OK-432 (as an adhesive agent) and minocycline into the bullae via the drainage tube to induce inflammatory adhesion. Of 2 patients treated with this approach, 1 showed partial efficacy. However, persistent air leakage occurred in 1 case, requiring intervention on postoperative day 37 for resolution. Our research team proposed an alternative approach involving intrabullous injection of erythromycin and hypertonic glucose (both acting as sclerosing agents^[25–27]^) into GEB to stimulate sterile inflammation. In our hypothesis, by continuously applying high negative pressure suction and repeatedly injecting a solidifying agent, the inflammatory response within the GEB can be maintained to solidify GEB and the bronchial wall for drainage, thereby achieving the reduction or closure of GEB. However, the challenge remains for GEB with abundant collateral ventilation, which may be difficult to shrink using the above method, accompanied by the risk of prolonged air leakage, thus complicating GEB solidifying and bonding. To address this challenge, selective bronchial occlusion by bronchoscopy was applied to block the target bronchus for drainage, thereby preventing persistent air leakage or its recurrence after treatment. The key technical difficulties in implementation were determining the target bronchus for drainage and selecting appropriate agents for closure.

When the patient was in the supine position, the erythromycin lactobionate solution injected into GEB via the drainage tube indeed tended to deposit dorsally due to gravitational effects. To address this, air extraction via the drainage tube was performed to rapidly reduce the GEB volume, causing the bulla to collapse toward its base. Following drug injection through the drainage tube, the majority of the drug deposits in the dorsal region of the GEB, while a small portion may accumulate at the bulla base and subsequently enter the lung parenchyma via the target bronchus (especially after positional changes), potentially causing drug-related pneumonia. In this case, after catheterization and aspiration of the GEB, the bulla shrank toward the right upper lobe, preliminarily localizing the target bronchus to the right upper lobe. Post-injection imaging confirmed drug deposition exclusively in the posterior segment of the right upper lobe. A follow-up CT scan 4 days later revealed that the reduced GEB remained localized to the right upper lung, with drug-related pneumonia observed only in the posterior segment of the right upper lobe (Fig. 1E). No drug deposition or pneumonia was detected in other regions of the right lung. These findings definitively identified the posterior segment of the right upper lobe as the target bronchus supplying the GEB.

The use of bronchoscopic autologous blood injection for GEB volume reduction has been preliminarily explored in previous studies. For instance, case reports^[28,29]^ have demonstrated transbronchial autologous blood injection as a feasible approach, achieving short-term therapeutic effects through blood clot-induced bronchial occlusion and subsequent bulla atrophy. Building on this, our study further optimized the strategy by incorporating thrombin to accelerate clot formation and expanding the injection scope to the entire affected lung lobe (when the precise target bronchus cannot be identified), aiming to comprehensively block GEB-associated collateral ventilation pathways, thereby addressing persistent air leakage – a common complication of percutaneous interventions. This cost-effective method leverages the inherent biological adhesive properties of blood, promoting bulla collapse via localized scar formation while minimizing reliance on expensive endobronchial devices. To enhance procedural efficacy, we integrated continuous negative-pressure drainage alongside bronchial occlusion. This dual approach facilitated sustained bulla adhesion and reduced residual airspace, as evidenced by rapid GEB closure on follow-up CT. Post-procedural absorption of blood-derived products allowed partial re-expansion of atelectatic lung tissue, contributing to functional recovery.

Furthermore, in contrast to bronchoscopic EBV placement, percutaneous bulla reduction demonstrates significant cost-effectiveness. Data from recent studies indicate that EBV placement imposes substantial economic strain, as each GEB patient typically requires implantation of 3 valves on average,^[6]^ with valve costs alone approaching $15,000 per procedure in China.^[18]^ This financial burden is particularly prohibitive in resource-limited settings.

## 4. Conclusion

This case report highlights the efficacy of a combined percutaneous and bronchoscopic approach for volume reduction in surgically ineligible patients with GEB. By integrating CT-guided aspiration, intrabullous sclerotherapy, and selective bronchial occlusion, this minimally invasive strategy achieved complete GEB closure, significant symptomatic improvement, and functional recovery in a high-risk patient with severe COPD. Notably, the use of percutaneous drug injection and aspiration proved instrumental in refining target bronchus identification when conventional imaging yielded ambiguous results, thereby enhancing procedural precision.

Despite these promising results, the study is limited by its single-case design and short-term follow-up. Larger prospective studies are warranted to validate long-term efficacy, optimize technical protocols, and identify patient subgroups most likely to benefit.

## Author contributions

**Conceptualization:** Lecong Ouyang, Zeqiang Wang.

**Formal analysis:** Lecong Ouyang, Weidong Zhang, Zeqiang Wang.

**Methodology:** Weidong Zhang, Zeqiang Wang.

**Writing – original draft:** Lecong Ouyang, Zeqiang Wang.

**Writing – review & editing:** Zeqiang Wang.
